# What it is like to be a bit: an integrated information decomposition account of emergent mental phenomena

**DOI:** 10.1093/nc/niab027

**Published:** 2021-11-16

**Authors:** Andrea I Luppi, Pedro A M Mediano, Fernando E Rosas, David J Harrison, Robin L Carhart-Harris, Daniel Bor, Emmanuel A Stamatakis

**Affiliations:** Division of Anaesthesia, School of Clinical Medicine, University of Cambridge, Cambridge CB2 0QQ, UK; Department of Clinical Neurosciences, University of Cambridge, Cambridge CB2 0QQ, UK; Leverhulme Centre for the Future of Intelligence, University of Cambridge, Cambridge CB2 1SB, UK; Department of Psychology, University of Cambridge, Cambridge CB2 3EB, UK; Center for Psychedelic Research, Department of Brain Science, Imperial College London, London W12 0NN, UK; Data Science Institute, Imperial College London, London SW7 2AZ, UK; Centre for Complexity Science, Imperial College London, London SW7 2AZ, UK; Leverhulme Centre for the Future of Intelligence, University of Cambridge, Cambridge CB2 1SB, UK; Department of History and Philosophy of Science, University of Cambridge, Cambridge CB2 3RH, UK; Center for Psychedelic Research, Department of Brain Science, Imperial College London, London W12 0NN, UK; Department of Psychology, University of Cambridge, Cambridge CB2 3EB, UK; Division of Anaesthesia, School of Clinical Medicine, University of Cambridge, Cambridge CB2 0QQ, UK; Department of Clinical Neurosciences, University of Cambridge, Cambridge CB2 0QQ, UK

**Keywords:** consciousness, information decomposition, integrated information theory, selfhood, phenomenology

## Abstract

A central question in neuroscience concerns the relationship between consciousness and its physical substrate. Here, we argue that a richer characterization of consciousness can be obtained by viewing it as constituted of distinct information-theoretic elements. In other words, we propose a shift from quantification of consciousness—viewed as integrated information—to its decomposition. Through this approach, termed Integrated Information Decomposition (ΦID), we lay out a formal argument that whether the consciousness of a given system is an emergent phenomenon depends on its information-theoretic composition—providing a principled answer to the long-standing dispute on the relationship between consciousness and emergence. Furthermore, we show that two organisms may attain the same amount of integrated information, yet differ in their information-theoretic composition. Building on ΦID’s revised understanding of integrated information, termed Φ_R_, we also introduce the notion of Φ_R_-ing ratio to quantify how efficiently an entity uses information for conscious processing. A combination of Φ_R_ and Φ_R_-ing ratio may provide an important way to compare the neural basis of different aspects of consciousness. Decomposition of consciousness enables us to identify qualitatively different ‘modes of consciousness’, establishing a common space for mapping the phenomenology of different conscious states. We outline both theoretical and empirical avenues to carry out such mapping between phenomenology and information-theoretic modes, starting from a central feature of everyday consciousness: selfhood. Overall, ΦID yields rich new ways to explore the relationship between information, consciousness, and its emergence from neural dynamics.

## Introduction

A central aim in both neuroscience and philosophy of mind is understanding the relationship between consciousness and its physical substrate. The past two decades have seen a proliferation of theoretical, experimental, and philosophical work on this topic (Crick and Koch 2003; Friston 2012; Tononi and Koch 2015; Koch *et.al.* 2016; Tononi *et al.* 2016; Parr and Friston 2019; Kleiner 2020). Additionally, an important and related question to how consciousness arises from matter concerns a mathematically precise and conceptually clear account of what constitutes ‘emergence’ (Tononi *et al.* 1998; Friston *et al.* 2014; Hoel *et al.* 2016; De Caro and Grasso 2017; Palacios *et al.* 2017, 2019; Hesp *et al.* 2019; Cea 2020). The present work is situated at the convergence of these two fundamental research avenues: i.e. in this paper we seek to contribute to the formalization of the relationship between consciousness and its underlying physical substrate and elucidate to what extent this relationship can be rigorously understood in terms of emergence. To this end, we demonstrate the value of leveraging novel information-theoretic approaches to shed light on the nature of consciousness.

Here, we understand consciousness to be that which is lost during dreamless sleep and deep anaesthesia and recovered upon awakening (Schwitzgebel 2016). Following William James’s celebrated notion of the stream of consciousness, we take the stance that subjective experiences dynamically unfold over time. For this reason, when seeking to understand the aspects of the physical world that correspond to subjective experiences, and give rise to them, we direct our attention to neural dynamics. Indeed, neural dynamics have recently been proposed as a ‘common currency’ between brain and mind (Northoff *et al.* 2020), and a growing body of empirical work has demonstrated—through a variety of neuroimaging modalities and analytic approaches—that considering neural dynamics can provide powerful insights into consciousness and its alterations (Barttfeld *et al.* 2015; Atasoy *et al.* 2017, 2018; Uhrig *et al.* 2018; Deco *et al.* 2019; Demertzi *et al.* 2019; Lee. *et al.* 2019; Lord *et al.* 2019; Luppi *et al.* 2019, 2020b, 2020c, 2021b; Vlisides *et al.* 2019; Dinesh *et al.* 2020; Huang *et al.* 2020; Standage *et al.* 2020; Varley *et al.* 2020a, 2020b; Cavanna *et al.* 2018; Cao *et al.* 2019; Cai *et al.* 2020; Eagleman *et al.* 2019; Hahn *et al.* 2021; Hutchinson *et al.* 2014).

Thus, both theoretical and empirical reasons lead us to adopt a process-theoretic framework to the study of consciousness. Shannon’s information theory is particularly well-suited to address process-theoretic approaches such as the one adopted here, because it enables the study of dynamical processes in a way that is not committed to any specific physical instantiation. This is not to say that the physicality of the system does not matter for the instantiation of consciousness: some material substrates may be more or less suitable (e.g. in terms of degrees of freedom) to sustain the dynamics on which consciousness depends (see esp. Barrett 2014). Rather, information theory enables us to focus on the dynamics and their properties, abstracted from considerations about physical instantiation or neuroimaging modality.

### From quantification of consciousness to its decomposition

The close relationship between consciousness and information is a key aspect of the well-known Integrated Information Theory of consciousness (hereafter, IIT) (Tononi *et al.* 1994; Balduzzi and Tononi 2008; Oizumi *et al.* 2014; Tononi *et al.* 2016; Tononi 2008 Manifesto). A core intuition from IIT is that the ‘integration of information’ represents a fundamental aspect of consciousness—and therefore, quantifying integrated information (suitably defined, as we explore in ‘Technical preliminaries’) enables an effective quantification of consciousness.

Quantification is a key step in the scientific process, and the notion that consciousness could be quantified has contributed in no small part to the scientific study of consciousness in the last two decades. After quantification, decomposition into fundamental elements can be a fruitful next step in the scientific process, driving major advances in fields such as physics and chemistry over the last two centuries. As an illustrative example, knowing whether the clear liquid in one’s glass is composed by twice as many hydrogen atoms as oxygen atoms, or oxygen and hydrogen in equal parts, represents the difference between drinking refreshing water or poisonous hydrogen peroxide. We believe that understanding the information-theoretic composition of consciousness will be similarly crucial for its scientific characterization.

Within this context, the aim of this paper is to shed light on consciousness from an information-theoretic perspective by capitalizing on major advances in information theory, specifically within the recent framework of Partial Information Decomposition (PID) (Bertschinger *et al.* 2014; Griffith and Koch 2014; Wibral *et al.* 2017; Lizier *et al.* 2018) and its multivariate extension, Integrated Information Decomposition (ΦID) (Mediano *et al.* 2021). Shannon’s seminal results on information theory are mainly focused on information transmission between a single source and a single target; PID extends classic information-theoretic approaches to systems involving multiple sources of information (Wibral *et al.* 2017). Moreover, the more recent development of ΦID further extends PID to scenarios involving multiple targets as well as multiple sources (Mediano *et al.* 2021). Such an extension is necessary if we are to investigate complex, multivariate systems with intricate causal relationships, of which the human brain is a prime example (Turkheimer *et al.* 2021; Varley *et al.* 2020b). Thus, information decomposition—and ΦID in particular—proves especially valuable for our purposes, being ideally suited to accommodate the multifaceted dynamics that characterize neural activity.

Here we propose a shift in perspective beyond the information-theoretic quantification of consciousness and towards its decomposition into fundamental information-theoretic elements. For this purpose, we explore the consequences of viewing the core intuition that consciousness can be quantified in terms of integrated information in the light of ΦID. Note that we are not thereby committed to any of IIT’s additional theoretical positions: for instance, we remain agnostic on whether integrated information is identical with consciousness or merely co-extensive with it under certain descriptions. Likewise, our approach does not deal with ‘composition’ in the IIT sense of which qualia contribute to a specific experience (see section: From bits to bats): rather, our focus is entirely on the notion of consciousness as quantifiable in terms of information and what the decomposition of this information can tell us about consciousness.

Overall, this paper has two key goals. First, to demonstrate that understanding the information-theoretic composition of consciousness may be crucial to obtain insights about what consciousness is—and the various modes in which it can be configured. Our second major goal is the formal demonstration, within the ΦID framework, that consciousness can comprise both emergent and non-emergent phenomena—which we demonstrate based on its information-theoretic composition.

We call this ΦID-based approach to consciousness ‘Psy-ID’ (ΨID), as it is the result of an application of ΦID to psychological phenomena. Thus, ΨID is a theoretical framework that seeks to understand consciousness and other mental phenomena using insights from the broadly applicable mathematics of information decomposition—and ΦID in particular. Importantly, this framework also sheds new light on other prominent theories of consciousness such as the Global Neuronal Workspace Theory (Dehaene and Changeuz 2011; Mashour *et al.* 2020) by providing an information-theoretic definition of the workspace in terms of synergistic interactions (Luppi *et al.* 2020b). In contrast to other theoretical accounts of consciousness, ΨID provides a formal framework that is applicable to neuroimaging data, which enables a range of practical investigations on such observed dynamics (Mediano *et al.* 2021; Luppi *et al.* 2020a). This is especially important because we take it as imperative to our theoretical endeavours to provide predictions about consciousness that can be tested—and possibly falsified—by our current neuroimaging methods.

The rest of this paper is structured as follows. First, ‘Technical preliminaries’ provides an accessible overview of the main information-theoretic intuitions and basic premises on which our work builds. Then, ‘Theoretical contributions’ introduces ΨID to explicate the ramifications of ΦID for understanding consciousness and the neural dynamics on which it depends. Therein, we specify core terminology that is relevant to the hypotheses and predictions we propose in the third section, ‘Empirical predictions’. In this final section, we explore testable hypotheses and predictions as applied to recent experimental work on psychedelics and consciousness. This paper concludes with a series of remarks on the implications of ΨID and its potential limitations.

## Technical preliminaries

This section develops the formal intuitions and basic premises behind IIT, from which our paper takes its start. We then follow with an elaboration of the PID framework and its extension to IIT, called ΦID.

### The mind’s Φ: integrated information theory

The starting point of our investigation is the notion that consciousness can be quantified in terms of the integrated information of a system—the fundamental intuition behind the early development of IIT (Tononi 2004; Balduzzi and Tononi 2008). Stemming from the seminal ideas put forward by Tononi *et al.* (1994), IIT’s core premise (which we share) is that the brain can be comprehensively characterized by its dynamics—i.e. the way in which the brain’s activity unfolds over time (Tononi and Edelman 1998; Northoff *et al.* 2020). Correspondingly, these dynamics allow the brain’s current state to contain some information about its past and future states. Mathematically, this information that ‘flows’ from the past to the future of a dynamical system can be captured by the so-called time-delayed mutual information (TDMI) (Barrett and Seth 2011), denoted as $I\left( {{X_t}; {X_{t + 1}}} \right)$, where ${X_t}$ and ${X_{t + 1}}$indicate the state of the system at consecutive times *t* and *t+*1. Broadly speaking, TDMI provides a first step towards quantifying the dynamical structure of a complex system (James *et al.* 2011).

A further refinement on TDMI is to consider not only the overall amount of information, but also its spatiotemporal distribution. Different parts of the brain exchange information constantly—and the existence and relevance of these precise informational patterns is uncontroversial in cognitive neuroscience. Moreover, although a precise, theoretically-based measure remains to be discovered, there is mounting evidence that the patterns of information that spread across the brain are related to consciousness (Casali *et al.* 2013; Luppi *et al.* 2020b).

One of the key insights of Tononi, Sporns, and Edelman was to realize that this information must be shared across the brain in particular ways, such that it is simultaneously integrated (so the brain can behave as a whole) and differentiated (so its parts can perform independent processing). These ideas materialized into specific formulae to quantify integration and differentiation in the brain. In particular, the first such quantity to explicitly include the brain’s dynamics into its formulation was the measure of integrated information initially proposed by Balduzzi and Tononi (2008) and subsequently revised to be applicable to experimental time-series data^1^ by Barrett and Seth (2011). For a given bipartition of ${X_t}$ into two parts $M_t^1, M_t^2$, the measure is given by

$$\begin{equation*}{{{\Phi}}^{WMS}} = I\left( {{X_t};\ {X_{t + 1}}} \right) - \mathop \sum \limits_{i = 1}^2 I\left( {M_t^i;\ M_{t + 1}^i} \right){\ }\end{equation*}$$.

As above, here ${X_t}$ denotes the state of the system as a whole at time *t*$M_t^i$ denotes the *i*^th^ part of *X* at time *t* (and likewise for ${M_{t + 1}} $ and $M_{t + 1}^i$). Thus, this measure compares the information flow between the past and the future (as captured by TDMI) observed in the whole system *X* with the flow observed within each of its parts—for this reason it is also referred to as ‘whole-minus-sum’ Φ (Mediano *et al.* 2021).^2^ This measure is easy to compute (compared with other Φ measures) (Oizumi *et al.* 2016) and represents a noteworthy attempt to capture the powerful intuitions behind IIT. However, once the original formulation from Balduzzi and Tononi is rendered suitable for practical empirical application (Barrett and Seth 2011;Barrett and Mediano 2019), the resulting mathematical formulation has known shortcomings, including the fact that it can yield negative values in some cases—which are hard to interpret, as a system cannot intuitively be ‘negatively integrated’ or have negative consciousness (Oizumi *et al.* 2016; Mediano *et al.* 2018). In the following, we show how these shortcomings can be overcome by means of the mathematical framework of ΦID.

Note that the formula for Φ^WMS^ above stems from what is known as IIT 2.0, but TDMI is by no means the only way of quantifying the dynamical structure of a system: indeed, subsequent developments in IIT 3.0 used alternative metrics with a more explicit focus on causal interpretations (Oizumi *et al.* 2014), which were in turn replaced in the latest iteration known as IIT 4.0 (Barbosa *et al.* 2020). Our rationale for following the quantification of integrated information from IIT 2.0 rather than these later versions is that IIT 2.0 is the last version of IIT to be within the scope of Shannon’s information theory. Since the goal of the present work is to investigate the potential of information decomposition to shed light on integrated information and consciousness (see section: Theoretical contributions), the more recent versions of IIT are not suitable for our purposes—thereby requiring us to work with the IIT 2.0 formulation of Φ.

### Information decomposition

The TDMI, on which Φ^WMS^ depends, is a special case of Shannon’s mutual information, *I.* On a conceptual level, this ‘information’ can be understood in several distinct but converging ways (Shannon 1948; Hellman and Raviv 1970; Feder and Merhav 1994; Jaynes 2003; Parrondo *et al.* 2015). While extremely versatile, Shannon’s information theory is (mainly) confined to interactions between pairs of variables (e.g. a sender and a receiver), and it is not equipped to study the information that multiple parts of a system have about each other’s dynamical evolution.

Part of the necessary technical equipment for such analysis was introduced by Williams and Beer (2010) with their framework of PID. PID, in short, decomposes the total information that two sources give about a target into four distinct ‘partial information atoms’, typically called ‘synergistic’, ‘redundant’, and ‘unique’ information [note that for the sake of simplicity, throughout this work we will only consider the case of bivariate systems: for details of generalization to more than two variables, we refer the reader to Mediano *et al.* (2021)].

As an illustrative example of redundant, unique, and synergistic information, consider humans’ two sources of visual information about the world: the eyes. The two eyes provide in part the same information, which is the information that is not lost when closing one or the other eye. This information is therefore carried redundantly by both sources. However, information about the very edge of the visual field is unique to each eye: this unique information is lost when the corresponding eye is closed. Finally, closing either eye will also remove stereoscopic information about depth: this information is not carried by either eye alone, but rather it requires both eyes together, arising synergistically from their interaction.

Thus, it is important to note that atoms are ways in which the information is being carried and should not be confused with the sources of information that are doing the carrying, nor with the specific content that is being carried: any reference to e.g. ‘synergy’ or ‘redundancy’ should be understood as shorthand for ‘information carried synergistically’ and ‘information carried redundantly’, and likewise for other atoms. Therefore, as a way of example, if both my eyes show me that there is a red apple in front of me, then each eye (source) is carrying redundantly (atom) information about an apple (target), with the information in question being that the apple is red (content).

By allowing us to distinguish qualitatively different phenomena involving multiple information sources that cannot be disentangled by classic information theory (Rosas *et al.* 2016), PID moves beyond Shannon’s information theory (James and Crutchfield 2017). These novel capabilities of PID have found fruitful application in multiple areas of neuroscience, from spike trains to whole-brain dynamics (Stramaglia *et al.* 2014; Wibral *et al.* 2017).

### Decomposing information flow

Thanks to the new lens provided by PID, it is now possible to decompose the information that the past state of each part of the system, $M_t^1$, $M_t^2$, carries about the future of the whole system, ${X_{t + 1}}$. Formally, for a system with two parts, PID states that the information that part *i* has about the future of the system can be decomposed as

$$\begin{equation*}I\left( {M_t^i;{X_{t + 1}}} \right) = Red\left( {{X_t};\ {X_{t + 1}}} \right) + U{n_i}\left( {{X_t};\ {X_{t + 1}}} \right)\vspace*{3pt}\end{equation*}$$

where $Red\left( {{X_t};\ {X_{t + 1}}} \right)$ is the so-called ‘redundant information’ provided by the sources (here, the parts of the system) and $U{n_i}\left( {{X_t};\ {X_{t + 1}}} \right)$ is the information that $M_t^i$ provides uniquely. Similarly, the joint mutual information (i.e. information that the past state of the system as a whole carries about its future) is decomposed as

$$\begin{align*} & I\left( {{X_t};\ {X_{t + 1}}} \right) = Red\left( {{X_t};\ {X_{t + 1}}} \right) + U{n_1}\left( {{X_t};\ {X_{t + 1}}} \right) + U{n_2}\left( {{X_t};\ {X_{t + 1}}} \right)\\
& \qquad \qquad \qquad + Syn\left( {{X_t};\ {X_{t + 1}}} \right)\vspace*{5pt}\end{align*}$$

**Figure 1.**
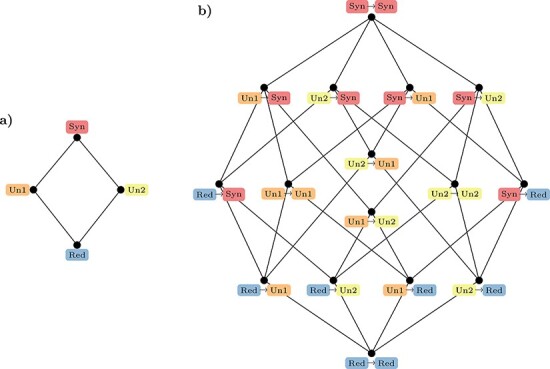
Visual representation of the relationships between PID and ΦID atoms for bivariate systems. In both cases, to aid visualization, atoms are arranged into a lattice, a mathematical construct which represents a hierarchical organization of information content, such that redundant information (held by every variable) is at the bottom and synergistic information (held only by the whole) is at the top. Please note that while here we focus in the case of two source variables, this information decomposition can be carried out over an arbitrary number of sources—which leads to a more elaborate lattice. For details of this more general construction we refer the reader to Williams (2011).

Here we see that in addition to redundancy and unique atoms, this expression includes $Syn\left( {{X_t}; {X_{t + 1}}} \right)$, which accounts for the so-called ‘synergistic’ information: information provided by both sources jointly, but not separately (Fig. 1a).

Thus, PID provides the means to understand how each part of the system carries information about the future state of the system as a whole, decomposing this information flow into redundant, unique and synergistic contributions. However, a key drawback of PID is that, while it is naturally applicable to multiple source variables, it can only be applied to scenarios with a single target. Thus, within the PID framework the future state of the system is still considered as a monolithic entity, without being able to consider the future state of each part individually. To resolve this shortcoming and extend the range of applicability of PID, Mediano *et al.* (2021) put forward the framework of ΦID, which forms the mathematical basis of our proposal. Formally, ΦID is a multi-target extension of PID that can identify the redundant, unique, and synergistic components of the information that multiple source variables carry about multiple target variables.

Specifically, the information that is carried in terms of each of the original four PID atoms (Red, Un_1_, Un_2_, and Syn, see Fig. 1a) at one point in time, at the next point in time may be carried as the same atom, or as any of the other atoms. Therefore, there are 4 × 4 = 16 combinations of PID atoms (Fig. 1b), which correspond to the decomposition established by ΦID. For instance, $U{n_1} \to Syn$ denotes the information that is initially carried uniquely by the first source and subsequently becomes carried synergistically. For more details about the interpretation of each of the ΦID atoms, and the generalization to more than two variables, we refer the reader to Mediano *et al.* (2021).

Taken together, PID and ΦID constitute valuable tools to refine our understanding of information processing in dynamical systems—and, therefore, can be used to refine our theories of any phenomenon that depends on such information processing, like consciousness. In the same way as PID allows us to refine our understanding of mutual information, we will see that ΦID allows us to refine our understanding of Φ^WMS^.

### Refining Φ through ΦID

ΦID makes it possible to decompose the information flow between the past and future states of each part of a system. Thus, a key feature of ΦID for our purposes is that it makes PID applicable to dynamical processes—including neural dynamics. A growing body of recent work has demonstrated that considering dynamical aspects of the brain can shed critical light on various aspects of consciousness (Northoff *et al.* 2020). For example, it has been shown that brain dynamics are significantly altered when consciousness is suppressed by anaesthesia or severe brain injury (Luppi *et al.* 2019; Luppi *et al.* 2021a; Huang *et al.* 2020; Demertzi *et al.* 2019; Barttfeld *et al.* 2015), or altered by psychedelics (Atasoy *et al.* 2017; Lord *et al.* 2019; Luppi *et al.* 2020c, 2021b). These results highlight the key role of neural dynamics for consciousness, vindicating the approach adopted by Tononi *et al.* (1998).

Specifically, ΦID allows us to decompose the total information flow from the past to the future [TDMI, introduced above as $I\left( {{X_t}; {X_{t + 1}}} \right)$] into a range of modes of information dynamics, which can be used to deepen our understanding of various dynamical phenomena observed in the brain. A deeper understanding of Φ^WMS^ and its drawbacks, such as the reasons why it can adopt negative values, can be gained by decomposing it into its constituent information atoms.

**Figure 2.**
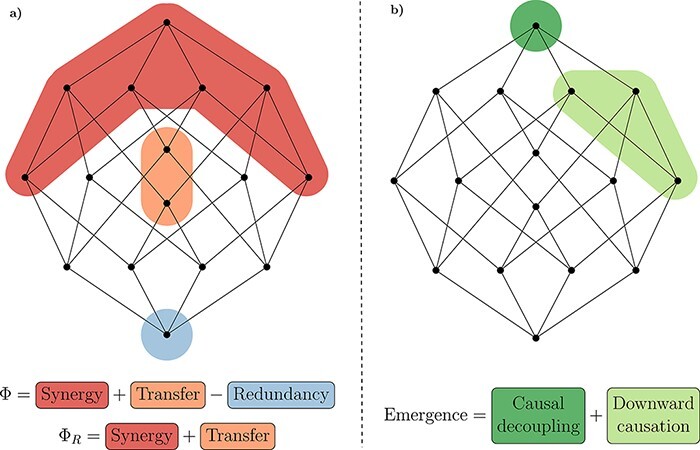
ΦID lattice with relevant atoms for Φ^WMS^ and Φ_R_ (**a**), and causal emergence (**b**) highlighted in colour.

Let us recall that ΦID decomposes the TDMI of a dynamical system with two parts into 16 disjoint atoms [illustrated in Fig. 1(b)]. Of those 16 atoms, it can be shown mathematically (Mediano *et al.* 2021) that 10 are represented in Φ^WMS^ (Fig. 2a): seven correspond to all the synergistic information in the system, two correspond to the information transferred from one part to the other, and, importantly, one redundancy atom with a negative sign. Through this decomposition of Φ^WMS^ one can understand why Φ^WMS^ can sometimes take negative values: the subtraction of the redundancy atoms implies that Φ^WMS^ will be negative in redundancy-dominated systems, whenever redundancy is greater than the sum of synergy and information transfer. Note that transfer and synergy are rather different phenomena: while transfer refers to information that ‘moves’ from one variable to another, synergy corresponds to phenomena that involve both variables, but cannot be seen in any of them when they are examined separately (Mediano *et al.* 2021).

Leveraging ΦID, a revised measure of integrated information, termed ‘Φ_R_’, can then be formulated by focusing on the synergy and transfer components and dropping the negative redundancy atom from the definition of integrated information (Mediano *et al.* 2021). Specifically, Φ_R_ is the sum of synergistic and transfer atoms, and it is thereby guaranteed to be non-negative. Thus, the information decomposition of Φ^WMS^ provided by ΦID can successfully identify the source of Φ’s theoretical difficulties and provide a straightforward solution. In addition to the theoretical improvement (which will be further discussed in Section ‘A tale of ice and Φ_R_: From neural dynamics to the Φ_R_-ing ratio’), there is also recent evidence that Φ_R_ also provides empirical advantages over the original formulation: unlike Φ^WMS^, Φ_R_ is reduced between the same sets of brain regions both in patients with chronic disorders of consciousness and during loss of consciousness induced by the intravenous anaesthetic, propofol (Luppi *et al.* 2020b). Importantly, reductions in Φ_R_ were reversed when participants recovered consciousness after anaesthesia, demonstrating the relevance of Φ_R_ for supporting human consciousness. Thus, given the theoretical advantages of Φ_R_ over Φ outlined here, as well as these recently demonstrated empirical benefits (Luppi *et al.* 2020b), we will consider Φ_R_ as our primary metric of integrated information and conscious level throughout the rest of this work.

### A deepness in the Φ: quantifying causal emergence

Another important feature of ΦID is that it provides a mathematical framework to build a formal, quantitative definition of causal emergence (Rosas *et al.* 2020a). Technically, the ΦID account of causal emergence rests on the following definitions. First, we say that a feature ${V_t}$ is ‘supervenient’ on the instantaneous state of the system at time *t* (denoted by ${X_t}$) if ${V_t}$ is a function of ${X_t}$, so that there is nothing about ${V_t}$ that can be predicted from the system’s previous state, ${X_{t - 1}}$, that cannot be already predicted from the system’s current state, ${X_t}$. Then, we say that a supervenient feature ${V_t}$ is ‘causally emergent’ if it has predictive power about the future evolution of ${X_t}$ that is unique (in the PID sense) with respect to the state of each part of the system.

Causal emergence is therefore defined here as the property of supervenient features to provide predictive power that cannot be explained from underlying microscale phenomena. In this manner, the coexistence of supervenience and irreducible predictive power of emergence—that have been previously thought as paradoxical (Bedau 1997, 2002)—can be resolved by operationalizing supervenience in terms of instantaneous relationships between the system and its features and identifying emergence with predictive power across time. Thus, a feature could be supervenient without being causally emergent, but not *vice versa*.

**Figure 3.**
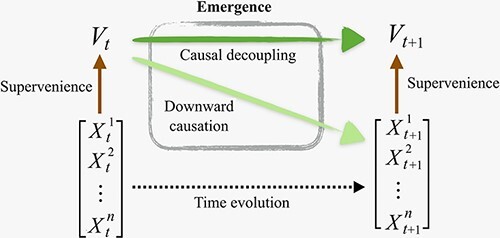
Causal decoupling and downward causation as two different types of emergent phenomena.

Crucially, the unique predictive power enabled by emergent features is quantified by ΦID. It is possible to prove that, under relatively general assumptions (Rosas *etal.* 2020b), a system’s capacity to host causally emergent features depends directly on how synergistic the system’s dynamics are. Furthermore, ΦID allows us to distinguish two qualitatively different types of emergence: ‘downward causation’, in which an emergent feature has unique predictive power over individual parts; and ‘causal decoupling’, in which it has unique predictive power not over any constituent, but only over the system as a whole (Figs 2b and 3).

Interestingly, causal decoupling corresponds to the persistent synergies in the system (top atoms in the ΦID lattice of Fig. 2b), which can be thought of as ‘the macroscale having causal influence on the macroscale, above and beyond the microscale effects’ (Rosas *et al.* 2020a) (Fig. 2b). Microscale and macroscale are then related by downward (macro-to-micro) causation, as well as upward (micro-to-macro) causation, which respectively correspond to transformations of information from and to synergy. The distinction between supervenience and emergence (i.e. causal decoupling and downward causation) is also illustrated in Fig. 3.

Note that the notion of causal emergence is not new, dating back at least to the book Problems of Life and Mind by George Lewes (Lewes 1879/2012; Turkheimer *et al.* 2021), with several proposed quantifications (see e.g. Bar-Yam 2004; Seth 2008), including in the context of IIT (Hoel *et al.* 2013, 2016). While the intervention-based approach of Hoel and colleagues has its own interest and domain of applicability, it does not fit well with our framework—which is not concerned with interventions, but is instead predicated in terms of information decomposition. Therefore, here we follow the approach introduced in Rosas *et al.* (2020a), while noting that future work may fruitfully combine these different approaches.

## Theoretical contributions: ΨID

To recapitulate, the aim of the present work is to provide a shift from the quantification of consciousness (viewed as integrated information) to its decomposition and demonstrate how understanding consciousness in terms of ΦID atoms can shed light on several aspects of consciousness, including its relationship with emergence. If the close relationship between mental and neural dynamics assumed here turns out to be correct, and integrated information (Φ_R_) happens to be a valid metric of conscious level, then our ΨID framework is ideally suited to provide insight on the role of the different ‘modes’ of neural dynamics—as determined by the ΦID atoms—in supporting various aspects of mental phenomena.

### Rising Φ_R_: quantifying consciousness as an emergent property

There has been abundant and vigorous philosophical debate over whether consciousness is an emergent phenomenon (Feinberg and Mallot 2020). By (i) understanding consciousness as integrated information, (ii) decomposing it into its constituent information-theoretic atoms through ΦID, and then (iii) viewing those atoms in terms of causal emergence, we are in a position to establish a grounded, formal, and falsifiable way to address this problem within the ΨID framework—representing the first core contribution of this paper.

In order to fully develop this argument, let us start noting that the ΦID decomposition of Φ_R_ in Fig. 2a and of emergence in Fig. 2b shows that consciousness (quantified by Φ_R_) comprises the atoms of emergence among its constituents, but also comprises additional atoms that are beyond the scope of emergence. The implications of this mathematical fact are profound and far-reaching. In effect, note that nothing in IIT or ΦID mandates that a given system should exhibit ‘all’ atoms that constitute Φ_R_ in order to be conscious: all that is required is the presence of at least one of Φ_R_’s constituent atoms with non-zero value. Following this rationale, we can draw two conclusions about the relationship between consciousness and emergence, based purely on a system’s information decomposition

1. Consciousness (integrated information) ‘can’ be an emergent phenomenon: there could be systems whose Φ_R_ derives (entirely or in part) from emergent ΦID atoms.
2. Consciousness ‘does not have’ to be emergent: there could also be systems whose Φ_R_ derives entirely from ΦID atoms that do not enter into the composition of emergence (e.g. information transfer or non-emergent synergy atoms).

Therefore, thanks to our information-decomposition approach to both consciousness and emergence, we can now see that the original question ‘Is consciousness an emergent phenomenon’ was fundamentally underspecified. Specifically, it was underspecified because neither consciousness nor emergence are monolithic constructs, but rather they are both constituted by multiple information-theoretic atoms, and—crucially—only some of the atoms that constitute integrated information are emergent. Once this is understood, the answer becomes clear: some possible kinds of consciousness are emergent, others are not, and others may comprise both emergent and non-emergent atoms—in a way that is amenable to empirical investigation.

### A tale of ice and Φ_R_: from neural dynamics to the Φ_R_-ing ratio

**Figure 4.**
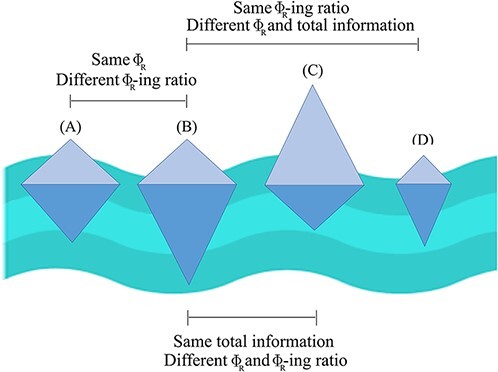
Differences in Φ_R_ and Φ_R_-ing ratio. Four conscious beings are schematically depicted as icebergs, with their total size representing their total information (TDMI), the part above water (light blue) representing the combination of atoms that compose Φ_R_ and the part below water being the information that does not contribute the Φ_R_ (and hence to consciousness). Different beings can have the same Φ_R_ despite different total information (A and B), or same total information but different Φ_R_ and Φ_R_-ing ratio (B and C), or different total information and different Φ_R_ but same Φ_R_-ing ratio (B and D). Only considering Φ_R_ would ignore the difference between A and B, as well as the similarity between B and D.

Decomposing Φ_R_ can also provide another important insight about the nature of consciousness. Since not all atoms enter into the composition of Φ_R_, dynamical processes with the same total amount of information flow (quantified by the TDMI) can still differ in their Φ_R_, and—conversely—organisms with the same Φ_R_ can differ in TDMI (Fig. 4). The proportion of TDMI that is accounted for by Φ_R_, which we may refer to as the ‘Φ_R_-ing ratio’, can then be thought as quantifying the efficiency with which the organism transforms information into consciousness—its ‘nougenic rate’. In other words, cognitive architectures can differ in the ‘consciousness bang’ that they provide for a given ‘informational buck.’

Note that the Φ_R_-ing ratio is in one respect closer than Φ_R_ to the original formulation of Φ, in terms of scaling negatively with redundancy. Original Φ^WMS^ explicitly removed redundancy from the quantification of integrated information, but this led to the theoretical flaw that the resulting quantity was not guaranteed to be lower-bound by zero and could instead be negative if redundancy dominated the system (see Section ‘Refining Φ through ΦID’). Being predicated as the ratio of Φ_R_ to TDMI, the Φ_R_-ing ratio implicitly takes redundancy into account, as a component of TDMI; in fact, the more redundancy there is in a system, the lower the Φ_R_-ing ratio will be even if Φ_R_ is high overall. In other words, both Φ^WMS^ and the Φ_R_-ing ratio acknowledge that there is an intuitive difference between a system where much of the information is redundant, and one that instead prioritizes dynamics related to consciousness (Φ_R_), and is therefore efficient in its ability to turn information into consciousness. The relationship between Φ and Φ_R_ is illustrated in Fig. 4.

At this stage we will not take a stance on whether the part of TDMI that does not contribute to Φ_R_ (e.g. the redundancy) is to be completely ignored from the study of consciousness—other than as contributing to the calculation of the Φ_R_-ing ratio—or whether perhaps it could be regarded as ‘preconscious’ or ‘potential consciousness’.

Thus, we argue that it can be beneficial to characterize information-processing systems with both Φ_R_ and Φ_R_-ing ratio, since they provide complementary views on integration information. Therefore, it should be clear that our proposed Φ_R_-ing ratio is not intended to replace Φ_R_, but rather to complement it, thereby enabling a more encompassing framework for quantifications of consciousness.

### Carved from Φ_R_: carving consciousness at its joints

Following a similar reasoning, an even more fundamental observation afforded by information decomposition is that even when two systems have the same Φ_R_ and the same Φ_R_-ing ratio, they may be entirely different in the composition of their Φ_R_. In fact, no single ΦID atom is essential for Φ_R_, suggesting that ‘two systems may have the same Φ_R_ despite not sharing any of its constituent atoms’ (e.g. a purely emergent system versus a transfer-only system). This possibility raises the fundamental question of whether different ΦID atoms may correspond to ‘different ways of being conscious’.

Implicitly, IIT is committed to an affirmative answer to this question: some ΦID atoms—it posits—are experienced and contribute to the system’s consciousness (integrated information), while other atoms are not: there is nothing it is like to instantiate those atoms (note that this is not the same as the problem of qualia; see section: From bits to bats). In contrast, ΨID suggests a richer perspective on this issue, by raising the possibility that different information-theoretic atoms may correspond to fundamentally different ‘ways of being conscious’—or, as we call them, ‘modes of consciousness’: broad-strokes aspects of how consciousness is subjectively experienced, which may be more or less extended in time.

Illustrative examples of specific modes of consciousness may include the selflessness experienced during deep meditation or under the effects of psychedelic drugs; or the general way that depression may feel to those suffering from it (see Colombetti and Ratcliffe 2012; Seth and Friston 2016; Deane 2020; Deane *et al.* 2020). Note also that although empirical evidence suggests that different cognitive functions rely on different information-theoretic atoms (Luppi *et al.* 2020a), our focus here is on altered states of consciousness, rather than normal fluctuations within day-to-day cognitive states.

In other words, ΨID contends that beyond the total amount of integrated information in an organism, additional insight may be gleaned from knowing its specific information-theoretic composition, which can be investigated through ΦID. By investigating the phenomenology associated with specific atoms, these ‘modes of consciousness’ may provide a way to eventually understand the phenomenology of different states of consciousness from the third-person perspective—as explained in the next section.

### From bits to bats

To demonstrate how ΨID can enrich our understanding of consciousness, we contend that if integrated information is an effective measure of conscious level, then the corollaries obtained from ΨID would bring us one step closer to addressing the famous conundrum that has dogged the philosophy of mind: ‘What it is like to be a bat’. That is, even though we elect to use the term ‘modes of consciousness’, and this term is different in its extension from the debate on qualia (for which Nagel’s (1974) is typically placed as the cornerstone), it may still allow progress in that matter.

The alchemists of old sought to turn lead into gold and failed mainly because their understanding of these substances reached only the level of their surface properties (shiny or dull, yellow, or grey). Today, thanks to our understanding of atomic composition, turning lead into gold via nuclear transmutation is feasible (although not financially wise). Likewise, ΨID may represent a similar change of reference frame for our understanding of consciousness: rather than talking of ‘human consciousness’ or ‘bat consciousness’ tout court (like alchemists conceived of gold and lead as ‘substances’), we propose that it may be a more proficuous avenue to consider their respective atomic constitutions in terms of ΦID and think in terms of information atoms rather than ‘molecules’. Put differently, rather than trying to perform the mental ‘human-to-bat’ conversion all at once, as Nagel proposed, an atomic understanding of integrated information may provide us with a Rosetta stone to guide a more nuanced comparison.

More generally, we claim that thinking in terms of ‘modes’ or ‘kinds’ of consciousness may be advantageous for the exploration of conscious states within, and potentially also across, organisms^3^. From this point of view, the work of Nagel may be seen as shaping the debate too narrowly, focusing the discussion on fine-grained aspects of being a bat (flying through the air, echolocating its prey, etc.), when it is unclear how much progress can be made from such a starting point towards truly understanding ‘what it is like to be a bat’. We contend that failure to solve the question on Nagel’s terms should not prescribe scientific pessimism, as it does not follow that ‘every’ aspect of bat consciousness is foreclosed from investigation. In fact, we claim that while the fine-grained details related to contents associated with certain qualia may be highly specific—and hence possibly incommensurable—one can still compare the broad-stroke modes. Indeed, if our approach proves viable, then there may be relevant inferences to be drawn in comparing quantified modes of consciousness via their corresponding ΦID atoms between different systems, which could lead to insightful phylogenetic or cognitive similarities between conscious creatures.

To illustrate this point, let us focus on the challenge of seeking a ΦID atomic decomposition of ‘what it is like to be a human experiencing an altered state of consciousness’, which can be thought as a first step paving a road towards addressing conscious experiences of other species. An atomic understanding of what it may be like to be in a given state of altered consciousness is based on three elements.

1. A description of the atomic composition of the integrated information generated by the human cognitive architecture under baseline conditions, to act as a reference point [although it should be noted that the question of defining a ‘baseline human state’ is itself not trivial, as even so-called ‘resting state’ may be better understood as ‘Random Episodic Spontaneous Thought’ (Breakspear *et al.* 2004)].
2. A description of the atomic composition of the integrated information generated in a particular state of interest. (If no Φ_R_ is generated, then the question would be solved with the trivial answer ‘nothing’, which might be the case for the alterations of consciousness induced by anaesthesia or disorders of consciousness, and indeed there is ongoing empirical research on this topic; see Luppi *et al.* 2020b).
3. An understanding of what kind of changes in consciousness may correspond to each observed change of the atoms that constitute Φ_R_.

**Figure 5.**
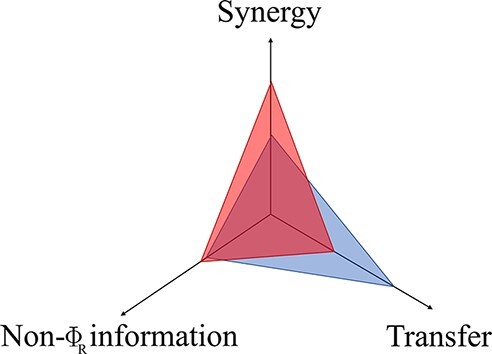
A low-dimensional representation of the information-theoretic atomic compositions of different systems. Each system (here, one shown in blue and the other in red) has a value for each of the three axes (which here summarize the full atomic composition). Synergy-containing atoms and information transfer are the two constituent elements of Φ_R_. The sum of a system’s projections on each axis represents its total information flow (TDMI).

Given these three pieces of information, we may be able to chart the atomic composition of baseline and altered human consciousness in a common ΦID space (an example of this is shown in Fig. 5).

The atomic composition of Φ_R_ may be found empirically by identifying an appropriate level at which neural information operates and then quantifying the prevalence of each atom in the human brain at baseline and during altered states of consciousness. While this endeavour is far from straightforward, there is at least a plausible understanding of how we may address this challenge. Importantly, while current neuroimaging techniques might not be able to provide a description at the desirable level of resolution, it is still possible for them to provide a trustworthy reflection of the lower level. Hence, the main challenge is finding out what synergy, transfer, and non-ΦR atoms are like, i.e. finding a phenomenological answer to ‘what it is like to be a bit’. The combination of ΦID with phenomenological interpretations of information-theoretic atoms is therefore the core of our proposed framework, ΨID.

Here we propose different (but potentially complementary) strategies that could be followed to identify which modes of consciousness could correspond to each of the atoms of Φ_R_. One avenue is to consider altered states of consciousness that one may expect to show an abundance—or lack of—specific atoms and understand how these differences might be reflected in the corresponding phenomenological alterations. These differences may be due to alterations induced by drugs (e.g. psychedelics and anaesthesia), neurological conditions, or even regular physiological alterations such as sleep, dreaming, or meditation. For instance, deep meditation and psychedelics such as lysergic acid diethylamide (LSD) can produce a state characterized by ‘loss of self’, or ‘ego dissolution’, and we may seek to identify which ΦID atoms or combination thereof correspond to this phenomenology by examining the information decomposition of neural signals during such states. Because this approach takes phenomenological evidence as a starting point, we refer to it as the ‘a posteriori’ ΨID approach.

An alternative approach to relate phenomenology and ΦID atoms is to try to identify what each atom may correspond to, in terms of subjective experience, based on what the atoms capture in terms of information processing. This may lead to predictions about how phenomenology should change if the atom in question were manipulated. Such predictions may then be tested empirically by studying perturbations that have been shown to alter the atomic composition [which may be discovered through the first (*a posteriori*) avenue described above]. Because it starts from abstract considerations in terms of information processing, we refer to this avenue as the ‘a priori’ approach of ΨID.

### What it is like to be a bit

At this point, it is worth clarifying an important difference between ΨID and IIT. Although for the present work we draw inspiration from IIT’s quantification of consciousness, IIT 3.0 and subsequent iterations also seek to address the qualitative character of consciousness (Oizumi *et al.* 2014). Briefly, IIT posits that each particular experience is identical with a so-called ‘maximally irreducible conceptual structure’ that is specified by a set of elements of a system, each being in a specific state and thereby jointly specifying a cause–effect structure for the system. Each quale (a ‘concept’ in the structure) specifies some qualitative aspect of the experience, based on what parts of the system’s cause–effect repertoire it constrains, which corresponds to phenomenal distinctions. In this context, the quantity of consciousness is intended to correspond to the distance between the conceptual structure specified by the state of the system as a whole and that specified by the system’s parts (i.e. its irreducibility). On the other hand, IIT posits that the geometry of the conceptual structure fully specifies the way that the experience feels—its qualitative character (Balduzzi and Tononi 2009; Tsuchiya *et al.* 2016; Moon and Pae 2019).

It follows that two specific experiences may have the same quantity of integrated information (i.e. they are equally irreducible), but different conceptual structures, corresponding to different phenomenal qualities (qualia: the redness of this particular apple; the painfulness of this specific instance of stubbing one’s toe). In this sense, IIT also goes beyond quantification, providing a conceptual account of how qualia contribute to a given experience (Albantakis et al. 2019; Haun and Tononi 2019; Albantakis and Tononi, 2019). However, this approach is fundamentally different from our own: IIT 3.0 aims to identify the phenomenological components of a specific experience. In contrast, ΨID’s modes of consciousness do not pertain to specific aspects of distinct individual experiences, but rather they are ways of experiencing, based on how the information is carried in the system. Thus, ΨID’s account in terms of modes of consciousness is more coarse-grained than the account of IIT 3.0: for instance, the contents of a given experience may change and that would change the qualia but may leave the mode the same (e.g. someone under the effects of LSD may be consistently experiencing the world through a low-synergy mode, regardless of what specific hallucination they are experiencing at a particular point in time; see Section ‘Me, Myself, and Φ’).

On the other hand, an advantage of our account is that it provides a full taxonomy of the information atoms that jointly constitute the information dynamics of any possible system—whereas we do not have a taxonomy of the fundamental elements that could compose any given conceptual structure (what IIT calls ‘qualia sensu stricto’), because each of them is specified in terms of a system’s cause–effect repertoire, and therefore the possible composition of each experience is not universal, but rather system-specific (i.e. species- or possibly even individual-specific). This means that although a bat may have qualia that are ‘alien’ to a human because of their different senses, the same information atoms may be found in principle both in the brain of a human and a bat, and this may provide avenues to relate human consciousness and bat consciousness. In other words, while the modes cannot tell us what a specific bat may be experiencing at a specific time (which the ‘conceptual structures’ of IIT 3.0 are allegedly intended to capture), nevertheless the modes may tell us what information processing in general (i.e. not pertaining to a specific content) is like for bats in general.

## Empirical predictions: Φnomenology

In the following sections, we outline how the theoretical framework of ΨID proposed here could function in practice, by conjecturing specific equivalences between ΦID atoms and various aspects of phenomenology and other psychological phenomena. These provisional predictions are empirically testable using current neuroimaging techniques and illustrate the practical value of ΨID.

At this point, it is worth recapitulating the extent and structure of our theoretical commitments. A fundamental assumption of this work is that it is theoretically possible and meaningful to quantify subjective experience (consciousness) in terms of integrated information of some (suitably chosen) neural dynamics. Subsequent to this background assumption, the core of our contribution is the hypothesis that it may be possible to obtain further insight into subjective experiences in terms of individual information-theoretic atoms, based on the ΦID framework.

### Me, myself, and Φ: self and persistent synergies

A fundamental aspect of phenomenology that may be understood in terms of ΨID is the self. Specifically, we propose that a system may constitute an individuated ‘self’ to the extent that its dynamics allow a persistent macroscale description of the system that is causally decoupled (as described in Section ‘A deepness in the Φ: Quantifying causal emergence’) from its individual constituent parts—here understood as the microscale. In other words, we posit that a self is associated with neural dynamics in which the macroscale has a causal influence on its own dynamics, beyond what is explainable from the individual microscale components. As discussed in ‘A deepness in the Φ: Quantifying causal emergence’, this notion corresponds to the presence of persistent synergies in the system, i.e. the ΦID atom of causal decoupling (Figs 2 and 3). The specific reason for identifying selfhood with causal decoupling is that only causal decoupling, among all ΦID atoms, identifies the emergence of a new causally efficacious macroscale entity that needs to be distinguished from the parts of the system, in order to obtain an accurate account of their joint future evolution. Note that these underlying intuitions are aligned with those of other recent works that conceptualize ‘being a self’in information-theoretic terms (Chang *et al.* 2020; Krakauer *et al.* 2020), although these works use mathematical frameworks that do not enjoy the expressive richness of ΦID (indeed, extension of these works with the ΦID framework would be an exciting avenue for future work).

Seen from this perspective, Hume’s account of a ‘bundle of perceptions’ (Hume 1986) ignores one fundamental issue: namely, that the parts together can constitute a whole beyond their sum, and high-order structures can persist even when the parts change—as has been formally demonstrated through ΦID (Rosas *et al.* 2020a).

Preliminary empirical support for a relationship between causal emergence (i.e. persistent synergy in the system) and the self is found in recent work, which suggests the brain’s default mode network to be highly synergistic (Luppi *et al.* 2020a). This association is promising, as the default mode network has been consistently implicated in self-referential processing (Qin and Northoff 2011) as well as the sense of self or ‘ego’ (Carhart-Harris and Friston 2010), and its integrity is compromised during LSD-induced ‘ego dissolution’ (Nour *et al.* 2016). Furthermore, the default mode network is also implicated in loss of consciousness, whether induced by anaesthesia or severe brain injury (Vanhaudenhuyse *et al.* 2010; Boveroux *et al.* 2010; Di Perri *et al.* 2017; Luppi *et al.* 2019, 2020b; Spindler *et al.* 2021). Indeed, the effect of general anaesthesia on the self is also a topic of recent interest (Sleigh *et al.* 2020). Thus, the DMN may constitute a nexus for the confluence of synergy, consciousness, and self in the human brain.

At this point, it is worth distinguishing the notions of ‘selfhood’ from ‘sense of self’. The former concerns the extent to which a system can be demarcated from its environment and stably exist as an independent entity—the extent to which that system constitutes an individual (Weber and Varela 2002). Following Krakauer *et al.* (2020), we take a system’s selfhood to be a graded property, which is determined by the system’s dynamics (Levin 2019). Accordingly, selfhood pertains to systems at all levels of organization, from humans to paramecia. In contrast, by ‘sense of self’ we refer to the extent to which an organism perceives ‘itself’ as having or being a self, which is related to higher-level metacognition [but see Friston (2018) for an alternative suggestion]. Therefore, a sense of self requires some degree of selfhood that can be acknowledged by the organism’s (possibly inaccurate) read-out of their own selfhood, whereas it is possible to have selfhood without a sense of self if the corresponding metacognitive abilities are not available. We leave open the question of whether any systems may, despite lacking selfhood, nevertheless come to have a delusional belief in their own selfhood—that is, having a sense of self without selfhood. Although seemingly unlikely and even paradoxical, our framework does not rule this out from a formal standpoint—and indeed, surprising and striking dissociations between belief and reality can be observed e.g. in patients suffering from anosognosia, who can adamantly deny obvious deficits from which they suffer.

Remarkably, although sense of self and consciousness seem tightly intertwined in everyday experience, evidence suggests that they can be dissociated. Both experienced meditators and people under the influence of psychedelics often report feeling a reduction in their experienced sense of self without acknowledging a diminution of their overall subjective experience—in fact, quite the opposite (Deane *et al.* 2020). The account of consciousness, individuality, and sense of self put forth by ΨID could accommodate both of these subjective experiences. Namely, this account would explain both the ‘self-less consciousness’ reportedly experienced by advanced meditators (Deane *et al.* 2020) and the ego dissolution induced by serotonergic psychedelics such as LSD and psilocybin (Carhart-Harris and Friston 2019) in terms of ΦR being preserved, but with a different information-theoretic composition that includes diminished levels of causal decoupling (Fig. 2)—corresponding to a state of consciousness with relatively reduced selfhood, which would then be metacognitively interpreted as reduced sense of self. An alternative hypothesis is that selfhood would be preserved (no reduction in causal decoupling), and the diminished sense of self would instead come about as a result of impaired read-out of the causal decoupling/selfhood—in other words, meditation and psychedelics would lead to illusions about one’s selfhood. These conjectures could be readily tested on magneto- or electro-encephalography or functional magnetic resonance imaging (fMRI) data, by determining whether reduced causal decoupling can be observed during the experiences in question.

**Figure 6.**
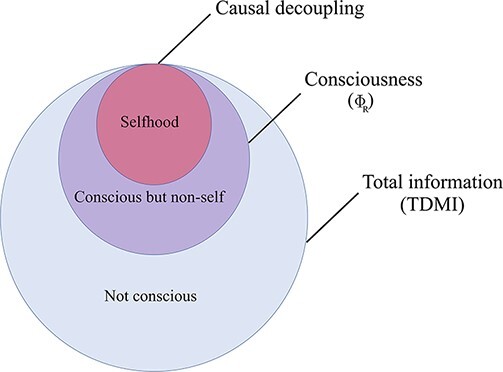
Visual representation of our hypothesized relationship between total information (large blue circle), consciousness (Φ_R_, medium purple circle), and selfhood (causal decoupling, violet small circle).

An important implication of identifying selfhood with the ΦID atoms that correspond to causal decoupling is that ‘all systems that have selfhood would also be conscious’—since causal decoupling atoms are among the consciousness-related atoms that compose Φ_R_. However, and perhaps surprisingly, ‘the converse is not the case: transfer-only systems may be conscious despite having no selfhood’, according to our proposed framework (see Fig. 6). This observation highlights the information-theoretic source of another kind of objection levelled against IIT, based on an intuitive resistance to the notion of certain systems (namely, transfer-only systems) being conscious. In effect, most of the counterexamples of synthetic systems with high Φ, and arguably no consciousness, display only transfer and no synergy (Oizumi *et al.* 2014).

Fortunately, decomposing Φ^WMS^ into its ΦID atoms allows us to bring this problem into focus, by formalizing it specifically in terms of whether or not information transfer should be considered as part of Φ^WMS^—whereas before the problem was merely about a collection of counterexamples. Prior to the information-decomposition approach, two answers were possible to the problem posed by these putative counterexamples: accepting the intuition behind them and rejecting tout court the idea that consciousness could be quantified in terms of Φ^WMS^; or rejecting the intuition. Our approach offers the possibility for a more nuanced, alternative middle ground: those who share the intuitions against transfer, but who also find appealing the intuitions at the core of IIT, now have the option of adopting (and trying to motivate) a transfer-free version of Φ^WMS^. This is analogous to the approach that we used to solve the problem of the negative values of Φ^WMS^: rather than simply rejecting Φ^WMS^ as a whole, we modified it to exclude the negative redundancy from its calculation, thereby obtaining ΦR. Of course, this type of solution may generalize beyond these particular counterexamples: other measures of integrated information may be defined from different combinations of atoms to suit specific theoretical needs. Thus, rather than using counterintuitive examples to reject the theory as a whole, we argue for refining the theory without throwing the baby out with the bathwater, by capitalizing on the finer-grained understanding made possible by ΦID.

### Zones of thought

One intriguing implication of the ΨID framework is that different environments may be conducive to the emergence of alternative modes of consciousness—just as they may be differently conducive to various forms of life. Most organisms harvest information about their environment through multiple sensory modalities. The various senses of these organisms often provide information that is, at least partly, about the same external stimuli. For example, the same event is often the cause of stimuli delivered by more than one modality, such as someone’s words being conveyed by their lip movements as well as their speech. This suggests that an environment where events tend to provide complementary information across sensory modalities can favour the presence of integration via synergy, and organisms that are able to exploit this property will likely find themselves at an advantage.

As an example, in a forest a red but not green round fruit is edible, but only when it smells sweet, so that edible exemplars are identifiable only when the two senses are combined. Such an environment may be expected to favour informational synergy between smell and sight in animals that rely on this fruit for nourishment. Conversely, animals whose main source of sustenance is easily identified by a single property (e.g. a specific colour or smell) may not benefit from synergy to the same degree. Thus, the neural architectures of different organisms may rely on different combinations of information-theoretic atoms, possibly shaped by their environment and evolutionary history—as suggested by recent empirical evidence demonstrating that human brains rely on synergy to a greater extent than macaque brains (Luppi *et al.* 2020a).

If, as we have argued, selfhood corresponds to causal decoupling (i.e. persistent synergies in the system), then environments that favour or discourage synergy may have an effect on selfhood (and presumably also sense of self, which here we propose to understand as an organism’s perception of its own selfhood). This prediction could be tested by means of virtual reality devices, which could be used to provide participants with visual, auditory, and tactile stimuli whose contents are entirely unrelated (i.e. stimuli that provide no synergy), and subsequently probe whether alterations have occurred in their sense of self, and also in the prevalence of ΦID atoms corresponding to causal decoupling in their neural dynamics, as measured e.g. with fMRI or other neuroimaging modality. Intriguingly, a multi-user virtual reality platform was recently found to produce ‘ego dissolution’ type experiences not incomparable to those produced by moderate doses of psychedelics (Glowacki *et al.* 2020).

Furthermore, as synergy derives from the advantage of combining different sensory sources, our theory predicts a degradation of selfhood as the unifying factor (and, presumably, the organism’s perception of its own selfhood, i.e. its sense of self) when there is a reduced benefit of integration between different sources of sensory information. In addition to modifying the structure of the environment, as proposed above, another way to reach a similar condition may be to add independent noise in each sensory modality. Doing so may be expected to result in a reduction of signal-to-noise ratio and, hence, a reduction in the proportion of information that each sensory source can contribute towards synergy. Further work will be required to make this hypothesis more specific; intriguingly, however, such a reduction in signal-to-noise ratio may be part of the mechanism by which LSD induces its well-known ego-dissolving effects—complementary of effects taking place in top-down activity (e.g. Carhart-Harris and Friston 2019). In effect, the 5-HT-2A agonistic properties of LSD lead to dysregulated spontaneous neuronal activity (Nutt *et al.* 2020), which may be expected to weaken the contingency between neuronal firing and external stimuli, hence introducing noise in each channel and reducing the ability of different sensory sources to provide synergistic information—corresponding to reduced causal decoupling, our proposed information-theoretic substrate for selfhood.

The converse of these hypotheses suggests that providing stimulation across different sensory modalities that allows for high synergy may reduce the extent of psychedelic-induced ego dissolution. Partial support to this hypothesis is provided by recent studies on the effect of different stimuli under LSD, which suggest a competition between the psychedelic effect of the drug and stimulus (Mediano *et al.* 2020). We predict that by increasing the synergy between different sensory sources through concurrent stimulation of multiple sensory modalities, one might reinforce the self-other boundary—and the subjective evaluation of it—resulting in diminished ego dissolution.

## Discussion

**Figure 7.**
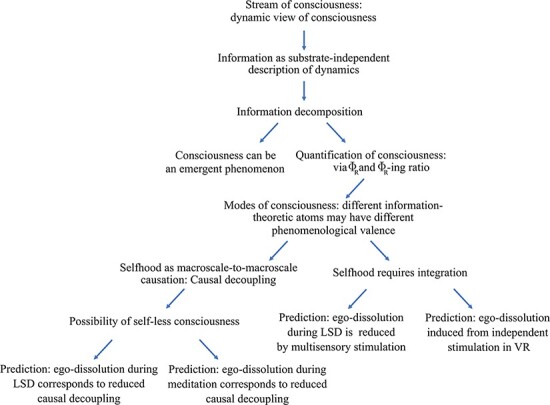
Visual summary of ΨID, illustrating the logical structure between the various claims made in this paper.

We have provided theoretical contributions about the nature of consciousness (understood as integrated information) based on its decomposition into elementary information-theoretic atoms and the key insight that the same total integrated information may be composed of different combinations of information atoms. This approach has allowed us to provide empirical predictions that could be tested with the tools of contemporary neuroscience. A schematic summary of our contributions, and the underlying assumptions, is provided in Fig. 7.

### To inΦnity and beyond

The main premise of this piece—and of ΨID more broadly—is that there is merit to the principle of quantifying consciousness via integrated information (suitably defined). We acknowledge that the predictions of ΨID outlined here are broad and high level (Fig. 7); however, we believe that it is important for a theoretical account to be clear about its empirical commitments. Indeed, we have made predictions in a broad range of areas—many of which could already be tested. Of course, there is still work to be done on finding the right temporal and spatial scale at which neural information should be best assessed and extending this framework to larger systems (e.g. based on recent developments of information decomposition that scale linearly with system size; Rosas *et al.* 2020b). Crucially, however, it is already possible to approach this quantification with effective results (see e.g. Luppi *et al.* 2020b).

Importantly, the advantages of a ΦID-based account of consciousness are not restricted to IIT, but rather this framework represents a general lens through which to understand consciousness. Indeed, the presented framework also provides insights about other theoretical approaches to consciousness, such as the Global Neuronal Workspace Theory (GNWT) (Dehaene and Changeuz 2011; Mashour *et al.* 2020). In effect, by re-thinking information exchange in the human brain in terms of synergy and redundancy, it is possible to delineate a ‘synergistic global workspace’ purely from functional considerations—based on brain regions whose connections are predominantly synergistic (Luppi *et al.* 2020b). Furthermore, analysis of resting-state fMRI data showed that loss of consciousness due to anaesthesia or brain injury corresponds to a reduction of Φ_R_ between regions of this synergistic workspace (more specifically, in the regions that regulate the entrance of information to the workspace, referred to as gateways by Luppi *et al.* 2020b). In this way, the more nuanced view on neural information processes offered by ΦID brings us closer to reconciling IIT’s and GNWT’s accounts of consciousness and offers the promise of further insights for our understanding of consciousness.

Another intriguing future research direction is to explore the relationship between the elaboration of ΨID explored herein and hierarchical predictive coding and the Free Energy Principle (FEP) (see Seth and Hohwy 2020). Interestingly, ideas in this direction have been put forward by Friston and colleagues in the context of IIT (Friston *et al.* 2020), where they suggest that—at least in terms of numerical analyses—the minimization of variational free-energy (which, according to the FEP, is a basic imperative for any self-maintaining cognitive system) ‘maximizes’ Φ and vice versa (Friston *et al.* 2020). Thus, an exciting question for future research is to assess how the notion of free-energy minimizing agents that also maximize Φ relates to our theoretical contributions put forward above: does maximization of Φ also correspond to maximization of Φ_R_ and how is it reflected in the Φ_R_-ing ratio? Is it driven by any specific ΦID atoms—such as synergy or transfer? Additionally, one could ask about the relationship between downward causation, discussed throughout our paper, to predictive mechanisms in a control hierarchy; specifically, where a higher-level component predicts some general feature of lower-level constituents. We thus believe there are promising overlaps between our account of ΨID and the theoretical ambitions of predictive coding and FEP approaches, which deserve to be explored in the future.

### So long, and thanks for all the Φs

The distinction of different ΦID atoms within integrated information leads to a new understanding of the space of possible consciousness, providing a phenomenological understanding of information-theoretic atoms: we call this framework ΨID. This framework provides not only a revised measure of integrated information, Φ_R_, but also a way to measure the efficiency of a system at turning information into consciousness—i.e. its ‘Φ_R_-ing ratio’. Importantly, when combined with a mathematical framework for the definition of causal emergence, ΨID enables us to propose a formal solution to the long-standing question of whether consciousness is an emergent phenomenon: namely, we show that both consciousness (understood as integrated information, Φ_R_) and emergence each comprise distinct sets of information-theoretic atoms, and only some of them overlap.

Thus, whether consciousness is emergent or not will depend on its information-theoretic composition. Moreover, since Φ_R_ is composed of different atoms, different systems may achieve the same amount of integrated information through different combinations of information-theoretic atoms. By investigating the phenomenology associated with specific atoms, these ‘modes of consciousness’ may provide a way to eventually understand the phenomenology of different states of consciousness that are not immediately accessible.

In addition to these theoretical contributions for a more nuanced understanding of consciousness, our ΨID framework also provides empirically testable predictions. In particular, we have hypothesized that ΦID atoms involving synergy may correspond to modes of being conscious that involve selfhood. We look forward to empirical tests of these predictions and the possible extension of our work to other mental phenomena, such as psychiatric conditions.

## Data Availability

Code to perform Integrated Information Decomposition is available upon request from author P.A.M.: email pam83@cam.ac.uk.

## Footnotes

1 The formulation of Φ in Balduzzi and Tononi requires a choice of reference distribution. The original choice of the maximum-entropy distribution, although theoretically attractive for a causal interpretation, is not always well-suited for practical applications: it is ill-defined for many systems of interest (Barrett and Mediano 2019); the transition probabilities from states that are rarely visited are difficult (if not impossible) to estimate accurately from empirical data; and the resulting measure may fail to capture a balance between integration and segregation—as one should expect from Φ-like measures (Mediano *et al*. 2021). Therefore, throughout this paper we adopt the ‘practical’ version of Φ proposed by Barrett and Seth (2011), which is based on the stationary distribution and addresses most of these issues.

2 Throughout the paper, and for consistency with prior literature (Mediano *et al*. 2018), we use the symbol Φ^WMS^ to refer to the specific quantity in the equation above and Φ to refer to the concept of integrated information in general.

3 We provide examples of this in Section ‘Me, Myself, and Φ’.

## References

[R1] Albantakis L , Marshall W, Hoel E et al. What caused what? A quantitative account of actual causation using dynamical causal networks. *Entropy* 2019;21:459. 10.3390/e21050459 PMC7514949 33267173

[R0001a] Albantakis L , Tononi G. Causal composition: structural differences among dynamically equivalent systems. *Entropy* 2019;21:1–29.doi: 10.3390/e21100989.

[R2] Atasoy S , Roseman L, Kaelen M et al. Connectome-harmonic decomposition of human brain activity reveals dynamical repertoire re-organization under LSD. *Sci Rep* 2017;7:17661. 10.1038/s41598-017-17546-0 PMC5732294 29247209

[R3] Atasoy S , Vohryzek J, Deco G et al. Common neural signatures of psychedelics: frequency-specific energy changes and repertoire expansion revealed using connectome-harmonic decomposition. *Prog Brain Res* 2018;242:97–120. 30471684 10.1016/bs.pbr.2018.08.009

[R4] Balduzzi D , Tononi G. Integrated information in discrete dynamical systems: motivation and theoretical framework. *PLoS Comput Biol* 2008;4:e1000091. 10.1371/journal.pcbi.1000091 PMC2386970 18551165

[R5] Balduzzi D , Tononi G. Qualia: the geometry of integrated information. *PLoS Comput Biol* 2009;5:e1000462. 10.1371/journal.pcbi.1000462 PMC2713405 19680424

[R6] Barbosa LS , Marshall W, Streipert S et al. A measure of intrinsic information. *Sci Rep* 2020;10:18803. 10.1038/s41598-020-75943-4 PMC7606539 33139829

[R7] Barrett AB . An integration of integrated information theory with fundamental physics. *Front Psychol* 2014;5:63. 10.3389/fpsyg.2014.00063 PMC3912322 24550877

[R8] Barrett AB , Mediano PAM. The phi measure of integrated information is not well-defined for general physical systems. *J Consciousness Stud* 2019;26:11–20.

[R9] Barrett AB , Seth AK. Practical measures of integrated information for time series data. *PLoS Comput Biol* 2011;7:e1001052. 10.1371/journal.pcbi.1001052 PMC3024259 21283779

[R10] Barttfeld P , Uhrig L, Stitt J et al. Signature of consciousness in the dynamics of resting-state brain activity. *Proc Natl Aca Sci* 2015;112:887–92. 10.1073/pnas.1418031112 PMC4311826 25561541

[R11] Bar-Yam Y . A mathematical theory of strong emergence using multiscale variety. *Complexity* 2004;9:15–24.

[R12] Bedau MA . Weak emergence. *Philos Perspec* 1997;11:375–99.

[R13] Bedau MA . Downward causation and the autonomy of weak emergence. *Principia: Int J Epistemology* 2002;6:5–50.

[R14] Bertschinger N , Rauh J, Olbrich E et al. Quantifying unique information. *Entropy* 2014;16:2161–83.

[R15] Boveroux P , Vanhaudenhuyse A, Bruno MA et al. Breakdown of within- and between-network rsting stake functional magnetic resonance imaging connectivity during propofol-induced loss of consciousness. *Anesthesiology* 2010;113:1038–53. 20885292 10.1097/ALN.0b013e3181f697f5

[R16] Breakspear M , Williams LM, Stam CJ. A novel method for the topographic analysis of neural activity reveals formation and dissolution of dynamic cell assemblies. *J Comput Neurosci* 2004;16:49–69. 14707544 10.1023/b:jcns.0000004841.66897.7d

[R17] Cai L , Wang J, Guo Y et al. Altered inter-frequency dynamics of brain networks in disorders of consciousness. *J Neural Eng* 2020;17:036006. 10.1088/1741-2552/ab8b2c 32311694

[R0017a] Cao B , Chen Y, Yu R et al. Abnormal dynamic properties of functional connectivity in disorders of consciousness. *NeuroImage* 2019;24.doi: 10.1016/j.nicl.2019.102071. PMC6881656 31795053

[R19] Carhart-Harris RL , Friston K. The default-mode, ego-functions and free-energy: a neurobiological account of Freudian ideas. *Brain: A J Neurol* 2010;133:1265–83. 10.1093/brain/awq010 PMC2850580 20194141

[R20] Carhart-Harris RL , Friston K. REBUS and the anarchic brain: toward a unified model of the brain action of psychedelics. *Pharmacol Rev* 2019;71:316–44. 31221820 10.1124/pr.118.017160 PMC6588209

[R21] Casali AG , Gosseries O, Rosanova M et al. Theoretically based index of consciousness independent of sensory processing and behavior. *Sci Transl Med* 2013;5:198ra105. 10.1126/scitranslmed.3006294 23946194

[R22] Cavanna F , Vilas MG, Palmucci M et al. Dynamic functional connectivity and brain metastability during altered states of consciousness. *NeuroImage* 2018;180:383–95. 28986208 10.1016/j.neuroimage.2017.09.065

[R23] Cea I . Integrated information theory of consciousness is a functionalist emergentism. *Synthese* 2020;1–26.doi: 10.1007/s11229-020-02878-832989333

[R24] Chang AYC , Biehl M, Yu Y et al. Information closure theory of consciousness. *Front Psychol* 2020;11:1–15.doi: 10.3389/fpsyg.2020.01504. 32760320 PMC7374725

[R25] Colombetti G , Ratcliffe M. Bodily feeling in depersonalization: a phenomenological account. *Emotion Rev* 2012;2:145–50.doi: 10.1177/1754073911430131.

[R26] Crick F , Koch C. A framework for consciousness. *Nat Neurosci* 2003;6:119–26. 12555104 10.1038/nn0203-119

[R27] De Caro M Grasso M . Three views on mental downward causation. In: Paoletti MP, Orilla F (eds), *Philosophical and Scientific Perspectives on Downward Causation*. New York: Routledge, 2017;313–27.

[R28] Deane G . Dissolving the self: active inference, psychedelics, and ego-dissolution. *Philosophy Mind Sci* 2020;1:1–27.doi: 10.33735/phimisci.2020.I.39.

[R29] Deane G , Miller M, Wilkinson S. Losing ourselves: active inference, depersonalization, and meditation. *Front Psychol* 2020;11:539726. 10.3389/fpsyg.2020.539726 PMC7673417 33250804

[R30] Deco G , Cruzat J, Cabral J et al. Awakening: predicting external stimulation to force transitions between different brain states. *Proc Natl Aca Sci* 2019;116:18088–97. 10.1073/pnas.1905534116 PMC6731634 31427539

[R31] Dehaene S , Changeuz JP. Experimental and theoretical approaches to conscious processing. *Neuron* 2011;70:200–27. 21521609 10.1016/j.neuron.2011.03.018

[R32] Demertzi A , Tagliazucchi E, Dehaene S et al. Human consciousness is supported by dynamic complex patterns of brain signal coordination. *Sci Adv* 2019;5:1–11.doi: 10.1126/sciadv.aat7603. PMC6365115 30775433

[R33] Di Perri C , Amico E, Heine L et al. Multifaceted brain networks reconfiguration in disorders of consciousness uncovered by co-activation patterns. *Hum Brain Mapp* 2017;39:89–103. 29024197 10.1002/hbm.23826 PMC6866397

[R34] Dinesh P , Li D, Dean JG et al. Level of consciousness is dissociable from electroencephalographic measures of cortical connectivity, slow oscillations, and complexity. *J Neurosci* 2020;40:605–18. 31776211 10.1523/JNEUROSCI.1910-19.2019 PMC6961988

[R35] Eagleman S , Chander D, Reynolds C et al. Nonlinear dynamics captures brain states at different levels of consciousness in patients anesthetized with propofol. *PLoS One* 2019;14:e0223921. 10.1371/journal.pone.0223921 PMC6821075 31665174

[R36] Feder M , Merhav N. Relations between entropy and error probability. *IEEE Trans Inf Theory* 1994;40:259–66.

[R37] Feinberg TE , Mallot J. Phenomenal consciousness and emergence: eliminating the explanatory gap. *Front Psychol* 2020;11:1041. 10.3389/fpsyg.2020.01041 PMC7304239 32595555

[R38] Friston K . A free energy principle for biological systems. *Entropy* 2012;14:2100–21. 23204829 10.3390/e14112100 PMC3510653

[R39] Friston K . Am I self-conscious? (Or does self-organization entail self-consciousness?) *Front Psychol* 2018;9:1–10.doi: 10.3389/fpsyg.2018.00579. 29740369 PMC5928749

[R40] Friston K , Sengupta B, Auletta G. Cognitive dynamics: from attractors to active inference. *Proc IEEE* 2014;102:427–445.

[R41] Friston KJ , Wiese W, Hobson AJ. Sentience and the origins of consciousness: from Cartesian duality to Markovian monism. *Entropy* 2020;22:516. 10.3390/e22050516 PMC7517007 33286288

[R42] Glowacki DR , Wonnacott MD, Freire R et al. Isness: using multi-person VR to design peak mystical type experiences comparable to psychedelics. In: CHI 20: Proceedings of the 2020 CHI Conference on Human Factors in Computing Systems. Honolulu, HI, USA: 2020. 1–14.doi: 10.1145/3313831.3376649.

[R43] Griffith V , Koch C. Quantifying synergistic mutual information. In: Prokopenko M. (eds) *Guided Self-Organization: Inception. Emergence, Complexity and Computation* vol 9. Berlin, Heidelberg: Springer, 2014. 10.1007/978-3-642-53734-9_6.

[R44] Hahn G , Zamora-Lopez G, Uhrig L et al. Signature of consciousness in brain-wide synchronization patterns of monkey and human fMRI signals. *NeuroImage* 2021;226:117470. 10.1016/j.neuroimage.2020.117470 33137478

[R45] Haun A , Tononi G. What does space feel the way it does? Towards a principled account of spatial experience. *Entropy* 2019;21:1160.

[R46] Hellman M , Raviv J. Probability of error, equivocation, and the Chernoff bound. *IEEE Trans Inf Theory* 1970;16:368–72.

[R47] Hesp C , Smith R, Parr T et al. Deeply felt affect: the emergence of valence in deep active inference. *Neural Comput* 2021;33:398–446.doi: 10.1162/neco_a_01341. 33253028 PMC8594962

[R48] Hoel EP , Albantakis L, Marshall W et al. Can the macro beat the micro? Integrated information across spatiotemporal scales. *Neurosci Consciousness* 2016;2016:1–12.doi: 10.1093/nc/niw012. PMC6367968 30788150

[R49] Hoel EP , Albantakis L, Tononi G. Quantifying causal emergence shows that macro can beat micro. *Proc Natl Aca Sci* 2013;110:19790–5. 10.1073/pnas.1314922110 PMC3856819 24248356

[R50] Huang Z , Zhang J, Wu J et al. Temporal circuit of macroscale dynamic brain activity supports human consciousness. *Sci Adv* 2020;6:eaaz0087. 10.1126/sciadv.aaz0087 PMC7065875 32195349

[R51] Hume D *A Treatise of Human Nature: Being an Attempt to Introduce the Experimental Method of Reasoning* . London: Penguin Classics, 1986.

[R52] Hutchinson RM , Hutchinson M, Manning KY et al. Isolurane induces dose-dependent alterations in the cortical connectivity profiles and dynamic properties of the brain’s functional architecture. *Hum Brain Mapp* 2014;35:5754–75. 25044934 10.1002/hbm.22583 PMC6869297

[R53] James RG , Crutchfield JP. Multivariate dependence beyond shannon information. *Entropy* 2017;19:531.

[R54] James RG , Ellison C, Crutchfield J. Anatomy of a bit: information in a time series observation. *Chaos* 2011;21:037109. 10.1063/1.3637494 21974672

[R55] Jaynes T *Probability Theory: The Logic of Science* . Cambridge: Cambridge University Press, 2003.

[R56] Kleiner J . Mathematical models of consciousness. *Entropy* 2020;22:609. 10.3390/e22060609 PMC7517149 33286381

[R0056a] Koch C , Massimini M, Boly M, Tononi G. Neural correlates of consciousness: progress and problems. *Nat Rev Neurosci* 2016;17:307–21. 27094080 10.1038/nrn.2016.22

[R57] Krakauer D , Bertschinger N, Olbrich E et al. The information theory of individuality. *Theory Biosci* 2020;139:209–23. 32212028 10.1007/s12064-020-00313-7 PMC7244620

[R58] Lee. H , Golkowski D, Jordan D et al. Relationship of critical dynamics, functional connectivity, and states of consciousness in large-scale human brain networks. *NeuroImage* 2019;188:228–38. 30529630 10.1016/j.neuroimage.2018.12.011

[R59] Levin M . The computational boundary of a “self”: developmental bioelectricity drives multicellularity and scale-free cognition. *Front Psychol* 2019;10:1–14.doi: 10.3389/fpsyg.2019.02688. 31920779 PMC6923654

[R60] Lewes HG . *Problems of Life and Mind*. Memphis, Tennessee: General Books LLC, 1879/2012.

[R62] Lizier J , Bertschinger N, Jost J et al. Information decomposition of target effects from multi-source interactions: perspectives on previous, current and future work. *Entropy* 2018;20:307. 10.3390/e20040307 PMC7512824 33265398

[R63] Lord LD , Expert P, Atasoy S et al. Dynamical exploration of the repertoire of brain networks at rest is modulated by psilocybin. *NeuroImage* 2019;199:127–42. 31132450 10.1016/j.neuroimage.2019.05.060

[R65] Luppi A , Craig M, Pappas I et al. Consciousness-specific dynamic interactions of brain integration and functional diversity. *Nat Commun* 2019;10:1–12. 31601811 10.1038/s41467-019-12658-9 PMC6787094

[R64] Luppi AI , Carhart-Harris R, Roseman L et al. LSD alters dynamic integration and segregation in the human brain. *NeuroImage*2021b;227:1–18.doi: 10.1016/j.neuroimage.2020.117653. PMC7896102 33338615

[R0065a] Luppi AI , Golkowski D, Ranft A et al. Brain network integration dynamics are associated with loss and recovery of consciousness induced by sevoflurane. *Hum Brain Mapp*2021a. 10.1002/hbm.25405. 10.1002/hbm.25405 PMC8127159 33738899

[R0065b] Luppi AI , Carhart-Harris RL, Roseman L et al. LSD alters dynamic integration and segregation in the human brain. *NeuroImage*2021b;227:117653.doi: 10.1016/j.neuroimage.2020.117653 PMC7896102 33338615

[R66] Luppi A , Mediano PAM, Rosas FE et al. A synergistic workspace for human consciousness revealed by integrated information decomposition. *bioRxiv*2020b. 10.1101/2020.11.25.398081. 10.7554/eLife.88173 PMC11257694 39022924

[R67] Luppi A , Mediano PAM, Rosas FE et al. A synergistic core for human brain evolution and cognition. *bioRxiv*2020a. 10.1101/2020.09.22.308981. 10.1038/s41593-022-01070-0 PMC7614771 35618951

[R68] Luppi A , Vohryzek J, Kringelbach ML et al. Connectome harmonic decomposition of human brain dynamics reveals a landscape of consciousness. *bioRxiv*2020c. 10.1101/2020.08.10.244459.

[R69] Mashour G , Roelfsema P, Changeux JP et al. Conscious processing and the global neuronal workspace hypothesis. *Neuron* 2020;105:776–98. 32135090 10.1016/j.neuron.2020.01.026 PMC8770991

[R70] Mediano PAM , Rosas FE, Luppi AI et al. Towards an extended taxonomy of information dynamics via Integrated Information Decomposition. *arXiv:2109.13186* 2021.

[R71] Mediano P , Seth AK, Barrrett AB. Measuring integrated information: comparison of candidate measures in theory and simulation. *Entropy* 2018;21:17. 10.3390/e21010017 PMC7514120 33266733

[R72] Mediano PAM , Rosas FE, Timmerman C et al. Effects of external stimulation on psychedelic state neurodynamics. *bioRxiv* 2020. 10.1101/2020.11.01.356071. 10.1021/acschemneuro.3c00289 PMC10853937 38214686

[R73] Moon K , Pae H. Making sense of consciousness as integrated information – evolution and issues of integrated information theory. *J Cognitive Psychol* 2019;20:1–52.

[R74] Nagel T . What is it like to be a bat? *Philos Rev* 1974;83:435–50.

[R75] Northoff G , Wainio-Theberge S, Evers K. Is temporo-spatial dynamics the ‘common currency’ of brain and mind? In quest of ‘spatiotemporal neuroscience. *Phys Life Rev* 2020;33:34–54. 31221604 10.1016/j.plrev.2019.05.002

[R76] Nour MM , Evans L, Nutt D et al. Ego-dissolution and psychedelics: validation of the Ego-Dissolution Inventory (EDI). *Front Hum Neurosci* 2016;10:1–13.doi: 10.3389/fnhum.2016.00269. 27378878 PMC4906025

[R77] Nutt D , Erritzoe D, Carhart-Harris RL. Psychedelic psychiatry’s brave new world. *Cell Commentary* 2020;181:24–8. 10.1016/j.cell.2020.03.020 32243793

[R79] Oizumi M , Albantakis L, Tononi G. From the phenomenology to the mechanisms of consciousness: integrated information theory 3.0. *PLoS Comput Biol* 2014;10:e1003588. 10.1371/journal.pcbi.1003588 PMC4014402 24811198

[R80] Oizumi M , Tsuchiya N, Amari S. Unified framework for information integration based on information geometry. *Proc Natl Aca Sci* 2016;113:14817–22. 10.1073/pnas.1603583113 PMC5187746 27930289

[R81] Palacios ER , Isomura T, Parr T et al. The emergence of synchrony in networks of mutually inferring neurons. *Sci Rep* 2019;9:1–14.doi: 10.1038/s41598-019-42821-7. 31040386 PMC6491596

[R82] Palacios ER , Razi A, Parr T et al. Biological self-organisation and Markov blankets. *bioRxiv* 2017;9:1–14.doi: 10.1101/227181.

[R83] Parr T , Friston K. Generalised free energy and active inference: can the future cause the past? *Biol Cybern* 2019;113:495–513. 31562544 10.1007/s00422-019-00805-w PMC6848054

[R84] Parrondo JMR , Horowitz JM, Sagawa T. Thermodynamics of information. *Nat Phys* 2015;11:131–9.

[R0084a] Qin P , Northoff G. How is our self related to midline regions and the default-mode network? *NeuroImage* 2011;57:1221–33.doi: 10.1016/j.neuroimage.2011.05.028. 21609772

[R85] Rosas F , Ntranos V, Ellison CJ et al. Understanding interdependency through complex information sharing. *Entropy* 2016;18:38.

[R86] Rosas FE , Mediano PAM, Rassouli B et al. An operational information decomposition via synergistic disclosure. *J Phys A: Math Theor*2020b;53:485001.

[R87] Rosas FE , Mediano PAM, Jensen JH et al. Reconciling emergences: an information-theoretic approach to identify causal emergence in multivariate data. *PLoS Comput Biol*2020a;16:e1008289. 10.1371/journal.pcbi.1008289 PMC7833221 33347467

[R88] Schwitzgebel E ’ Phenomenal consciousness, defined and defended as innocently as i can imagine. *J Consciousness Stud* 2016;23:224–35.

[R90] Seth A . Measuring emergence via nonlinear Granger causality. *Artif Life* 2008;11:545. 10.1162/artl.2010.16.2.16204 20067405

[R91] Seth A , Friston K. Active interoceptive inference and the emotional brain. *Philos Trans R Soc B* 2016;371:1–10.doi: 10.1098/rstb.2016.0007. PMC5062097 28080966

[R92] Seth AK , Hohwy J. Predictive processing as an empirical theory *for* consciousness science. *Cognitive Neurosci* 2020;12:1–2.doi: 10.1080/17588928.2020.1838467. 33280521

[R93] Shannon CE . A mathematical theory of communication. *Bell System Technical J* 1948;27:379–423, 623–656.

[R94] Sleigh J , Warnaby C, Tracey I. General Anaesthesia as fragmentation of selfhood: insights from electroencephalography and neuroimaging. *Br J Anaesthesia* 2020;121:233–40. 10.1016/j.bja.2017.12.038 29935577

[R0094a] Spindler LRB , Luppi AI, Adapa RM, Craig MM et al. Dopaminergic brainstem disconnection is common to pharmacological and pathological consciousness perturbation. *Pro Nat Acad Sci* 2021;118:e2026289118. 10.1073/pnas.2026289118 PMC8325270 34301891

[R95] Standage D , Areshenkoff CN, Nashed JY et al. Dynamic reconfiguration, fragmentation, and integration of whole-brain modular structure across depths of consciousness. *Cereb Cortex* 2020;30:5229–41. 32469053 10.1093/cercor/bhaa085 PMC7472202

[R96] Stramaglia S , Cortes JM, Marinazzo D. Synergy and redundancy in the Granger causal analysis of dynamical networks. *New J Phys* 2014;16:105003.

[R97] Tononi G . An information theory of consciousness. *BMC Neurosci* 2004;5:42. 10.1186/1471-2202-5-42 PMC543470 15522121

[R98] Tononi G . Consciousness as integrated information: a provisional manifesto. *Biol Bulletin* 2008;215:216–42. 10.2307/25470707 19098144

[R99] Tononi G , Boly M, Massimini M et al. Integrated information theory: from consciousness to its physical substrate. *Nat Rev Neurosci* 2016;17:450–61. 27225071 10.1038/nrn.2016.44

[R100] Tononi G , Edelman G. Consciousness and complexity. *Science* 1998;282:1856–1851. 10.1126/science.282.5395.1846 9836628

[R101] Tononi G , Koch C. Consciousness: here, there, and everywhere? *Philos Trans R Soc B* 2015;370:20140167. 10.1098/rstb.2014.0167 PMC4387509 25823865

[R102] Tononi G , Sporns O, Edelman G. A measure for brain complexity: relating functional segregation and integration in the nervous system. *Proc Natl Acad Sci USA* 1994;91:5033–7. 8197179 10.1073/pnas.91.11.5033 PMC43925

[R103] Tononi G , Sporns O, Edelman G. Complexity and coherency: integrating information in the brain. *Trends Cognitive Sci* 1998;2:474–84. 10.1016/s1364-6613(98)01259-5 21227298

[R104] Tsuchiya N , Taguchi S, Saigo H. Using category theory to assess the relationship between consciousness and integrated information theory. *Neurosci Res* 2016;107:1–7. 26748074 10.1016/j.neures.2015.12.007

[R105] Turkheimer FE , Rosas FE, Dipasquale O et al. A complex systems perspective on neuroimaging studies of behaviour and its disorders. *Complex Systems Perspective on Neuroimaging Studies of Behaviour and Its Disorders Preprints* 2020.doi: 10.20944/preprints202008.0654.v1.

[R0105a] Turkheimer FE , Rosas FE, Dipasquale O et al. A complex systems perspective on neuroimaging studies of behavior and its disorders. *Neuroscientist* 2021. 10.1177/1073858421994784. 10.1177/1073858421994784 PMC9344570 33593120

[R106] Uhrig L , Stitt JD, Jacob A et al. Resting-state dynamics as a cortical signature of anaesthesia monkeys. *Anesthesiology* 2018;129:942–58. 30028727 10.1097/ALN.0000000000002336

[R107] Vanhaudenhuyse A , Noirhomme Q, Tshibanda LJ-F et al. Default network connectivity reflects the level of consciousness in non-communicative brain-damaged patients. *Brain: A J Neurol* 2010;133:161–71. 10.1093/brain/awp313 PMC2801329 20034928

[R108] Varley TF , Luppi A, Pappas I et al. Consciousness & brain functional complexity in propofol anaesthesia. *Sci Rep*2020b;10:1018. 10.1038/s41598-020-57695-3 PMC6978464 31974390

[R109] Varley TF , Denny V, Sporns O et al. Topological analysis of differential effects of ketamine and propofol Anesthesia on brain dynamics. *R Soc Open Sci* 2021;8:201971.doi: 10.1098/rsos.201971. PMC8220281 34168888

[R110] Vlisides PE , Li D, Zierau M et al. Dynamic cortical connectivity during general anesthesia in surgical patients. *Anesthesiology* 2019;130:885–97. 30946057 10.1097/ALN.0000000000002677 PMC6520139

[R111] Weber A , Varela J. Life after Kant: natural purposes and the autopoietic foundations of biological individuality. *Phenomenol Cognit Sci* 2002;1:97–125.

[R112] Wibral M , Priesemann V, Kay JW et al. Partial information decomposition as a unified approach to the characterization and design of neural goal functions. *Brain Cognition* 2017;112:25–38.doi: 10.1016/j.bandc.2015.09.004. 26475739

[R113] Williams PL . Information dynamics: its theory and application to embodied cognitive systems. Ph.D. Thesis, Indiana University, 2011.

[R114] Williams PL , Beer RD. Nonnegative decomposition of multivariate information. *arXiv:1004.2515* 2010.

